# Biochemical and biophysical investigations of the interaction between human glucokinase and pro-apoptotic BAD

**DOI:** 10.1371/journal.pone.0171587

**Published:** 2017-02-09

**Authors:** Alix Rexford, Diego A. R. Zorio, Brian G. Miller

**Affiliations:** Department of Chemistry and Biochemistry, Florida State University, Tallahassee, Florida, United States of America; Russian Academy of Medical Sciences, RUSSIAN FEDERATION

## Abstract

The glycolytic enzyme glucokinase (GCK) and the pro-apoptotic protein BAD reportedly reside within a five-membered complex that localizes to the mitochondria of mammalian hepatocytes and pancreatic β-cells. Photochemical crosslinking studies using a synthetic analog of BAD’s BH3 domain and *in vitro* transcription/translation experiments support a direct interaction between BAD and GCK. To investigate the biochemical and biophysical consequences of the BAD:GCK interaction, we developed a method for the production of recombinant human BAD. Consistent with published reports, recombinant BAD displays high affinity for Bcl-xL (K_D_ = 7 nM), and phosphorylation of BAD at S118, within the BH3 domain, abolishes this interaction. Unexpectedly, we do not detect association of recombinant, full-length BAD with recombinant human pancreatic GCK over a range of protein concentrations using various biochemical methods including size-exclusion chromatography, chemical cross-linking, analytical ultracentrifugation, and isothermal titration calorimetry. Furthermore, fluorescence polarization assays and isothermal titration calorimetry detect no direct interaction between GCK and BAD BH3 peptides. Kinetic characterization of GCK in the presence of high concentrations of recombinant BAD show modest (<15%) increases in GCK activity, observable only at glucose concentrations well below the *K*_0.5_ value. GCK activity is unaffected by BAD BH3 peptides. These results raise questions as to the mechanism of action of stapled peptide analogs modeled after the BAD BH3 domain, which reportedly enhance the *V*_max_ value of GCK and stimulate insulin release in BAD-deficient islets. Based on our results, we postulate that the BAD:GCK interaction, and any resultant regulatory effect(s) upon GCK activity, requires the participation of additional members of the mitochondrial complex.

## Introduction

Glucokinase (GCK) catalyzes the rate-limiting step of glycolysis in β-cells and hepatocytes, where it regulates glucose-stimulated insulin secretion (GSIS) and glycogen synthesis, respectively [1,2]. Genetic deficiencies in GCK are associated with maturity onset diabetes of the young type 2 (MODY-II) and permanent neonatal diabetes mellitus [3,4]. Conversely, gain-of-function mutations in *gck* result in persistent hypoglycemic hyperinsulinemia of infancy (PHHI) [5]. GCK displays positive cooperativity and a high glucose *K*_0.5_ value, two features that allow this enzyme to be highly responsive to small changes in physiological glucose levels [6]. Recently, GCK was found to co-localize with the pro-apoptotic protein BAD in a 232 kDa complex at the mitochondrial membrane of hepatocytes and pancreatic β-cells [7]. Proteomic analysis of the complex identified additional protein components, including the catalytic subunit of protein kinase A (PKA), protein phosphatase 1 (PP1), and the actin-modifying, A kinase anchoring protein WAVE-1. The discovery of a complex that contains both BAD and GCK, along with the observation that BAD-deficient mice display glucose homeostatic dysfunction, led to speculation that glycolytic metabolism and apoptosis might be coordinated by BAD in the pancreas and liver [7].

Genetic and physiological studies of BAD-knockout mice demonstrate that the mitochondrial complex is crucial to proper β-cell function and hepatic metabolism. The mitochondrial complex fails to form in the absence of BAD, resulting in reduced glucose-driven oxygen consumption and impaired GSIS in β-cells [8]. BAD-deficient β-cells also exhibit decreased hyperpolarization of the mitochondrial membrane potential and dysfunctional [Ca^2+^]_i_ response. Notably, adenovirus-mediated reintroduction of BAD into BAD-deficient islets restores GSIS. In hepatocytes, BAD deficiency results in insulin resistance, as well as elevated gluconeogenesis and impaired glycolysis, a phenotype that is reproduced by *gck* knockdown [9]. The similar metabolic consequences of BAD and GCK depletion suggest that these proteins form a functional unit capable of regulating hepatic glucose metabolism.

Phosphorylation of BAD modulates its participation in apoptosis and glucose metabolism. High glucose concentrations stimulate BAD phosphorylation at three serine residues, resulting in reduced pro-apoptotic activity. In particular, phosphorylation at Ser155, within the BH3 domain of murine BAD, prevents interaction with Bcl-xL, thereby quenching BAD’s apoptotic activity [10–12]. Although the phosphorylation state of BAD does not impact formation of the GCK-containing mitochondrial complex, mice expressing a non-phosphorylatable S155A BAD variant display impaired GSIS [7,8]. Moreover, interference with Ser155 phosphorylation promotes fasting hyperglycemia and gluconeogenesis in the liver [9]. In BAD-knockout hepatocytes, normal glycolytic metabolism can be restored by the introduction of an S155D phosphomimic BAD variant, but not by the S155A analog. The S155D variant also stimulates GCK activity in hepatocytes, an observation that has led to the suggestion that GCK is the vehicle through which BAD acts to modulate glucose homeostasis [9].

Several lines of evidence support a direct interaction between GCK and BAD. *In vitro* transcription/translation of the two proteins indicates that BAD can coimmunoprecipitate with GCK in rabbit reticulocyte lysates [8]. In addition, a photoactivatable stapled peptide, designed to mimic the α-helical BH3 domain of BAD, cross-links to GCK upon UV exposure [13]. Mass spectrometry analysis of GCK cross-linked to the stapled phosphopeptide suggests that it associates with GCK near the active site, at a location distinct from the binding site of other known synthetic activators. Kinetic assays reveal that BAD BH3 stapled peptides increase GCK *V*_max_ by 29–44%, with little effect on cooperativity or the glucose *K*_0.5_ value [13]. The importance of specific BH3 residues for the GCK interaction is evidenced by the observation that substitution of alanine for conserved BH3 residues Leu-151, Ser-155, and Asp-156 reduces GCK association. Interestingly, stapled peptide BH3 analogs are also capable of restoring the impaired insulin response of BAD-deficient islets [8].

Our lab is focused on elucidating the biochemical mechanisms by which GCK activity is regulated. Past studies have demonstrated that GCK undergoes a slow, glucose-mediated conformational transition that is responsible for its unique allosteric properties, and which facilitates its central role in glucose homeostasis [14–16]. Activation of GCK, via either PHHI-associated disease variants or small-molecule therapeutic agents, involves alteration in the conformational landscape of GCK in favor of an ordered, more active enzyme conformation [17]. To investigate whether the previously reported BAD-mediated activation of GCK shares a similar mechanism, we produced full-length recombinant BAD and investigated the kinetic, thermodynamic, and structural impact of its interaction with recombinant human pancreatic GCK. In contrast to prior reports however, our investigation indicates that isolated full-length BAD and GCK do not interact *in vitro*.

## Materials and methods

### Cloning, expression, and purification of proteins

Recombinant human pancreatic GCK containing an N-terminal hexahistidine tag was produced in the glucokinase auxotroph BM5340(DE3) and purified using a previously described procedure [18]. TEV protease was expressed as a GST-fusion protein from a pGEX plasmid encoding the S219V variant, provided by David Waugh (Addgene plasmid 8827) [19]. Expression was induced with IPTG (1 mM) for 4 h at 30°C. Cell pellets were resuspended in Tris buffer (50 mM, pH 8.0) containing NaCl (100 mM), glycerol (10% v/v), benzamidine (1 mM) and dithiothreitol (10 mM) and lysed via French Press. Polyethyleneimine (0.1%) was added to the lysate and protein was purified on a glutathione-affinity column (GSTrap, GE Sciences). TEV protease was flash frozen and stored at -80°C for up to 1 month.

A pET15(b) based plasmid containing the catalytic subunit of protein kinase A (PKAc) cDNA was kindly provided by Susan Taylor (Addgene plasmid 14921) [20]. PKAc was expressed in BL21(DE3) and purified as previously described [21]. Protein was dialyzed against Tris buffer (100 mM, pH 8.0) containing KCl (100 mM), EDTA (1 mM), glycerol (10% v/v) and 2-mercaptoethanol (5 mM). PKA was flash frozen and stored at -80°C for up to 3 months.

Exon 1 of human *bcl-xl* lacking the C-terminal residues 189–233 was obtained from Stanley Korsmeyer (Addgene plasmid 8755) and site-directed mutagenesis was performed to create the Δ45–84, Δ210–233 variant [22]. Bcl-xL was expressed and purified using a modified procedure described previously [23]. Briefly, Bcl-xL was purified by nickel affinity chromatography and dialyzed against potassium phosphate buffer (20 mM, pH 7.4) containing NaCl (50 mM), EDTA (1 mM), and DTT (10 mM). Protein was applied to a Superdex 200 10/30 HR size-exclusion column, at a flow rate of 0.02 mL/min, to remove aggregate and oligomers prior to binding assays.

The 507 bp *bad* cDNA was amplified from Image Consortium CloneID 3537915 [24]. The cDNA was ligated into pET28(b) encoding an N-terminal hexahistidine tag and a C-terminal GST fusion with a TEV consensus sequence between the *bad* and the *gst* sequence. pET28*bad-gst* was transformed into BL21(DE3) and grown to mid-log phase. Expression was induced with IPTG (0.1 mM) for 4 h at 37°C. Cell pellets were then subjected to both native and denaturing purification protocols.

Under native conditions, cell pellet was resuspended and lysed in HEPES buffer (50 mM, pH7.6) containing NaCl (50 mM), imidazole (25 mM), glycerol (5%), and DTT (5 mM). PMSF, benzamidine, a protease inhibitor cocktail (Pierce), and BSA (0.1 mg/mL) were added to the lysis buffer in attempts to reduce protein degradation. Clarified lysate was applied to a HisTrap affinity column and following purification, purified BAD-GST was applied to a Superdex 200 10/30 HR size-exclusion column, at a flow rate of 0.02 mL/min.

Under denaturing conditions, the cell pellet was resuspended in lysis buffer containing sodium phosphate (0.1 M, pH 8.0), urea (6 M) and Tris-HCl (10 mM). Clarified lysate was subjected to nickel-affinity chromatography, and bound proteins were eluted with lysis buffer adjusted to pH 4. The sample was then dialyzed against refolding buffer (100 mM Tris-HCl, pH 8.0, 100 mM KCl, 10% glycerol, 5 mM DTT, and additives described below) containing urea in decreasing concentrations such that the buffer contained no urea after seven buffer changes over 36 h. Refolded protein was subjected to glutathione-affinity chromatography to remove improperly folded protein. Protein was eluted from the column using refolding buffer containing glutathione (10 mM) and was dialyzed against refolding buffer to remove glutathione. The sample was subjected to size-exclusion chromatography on a Superdex 200 10/30 HR column (0.02 mL/min), to separate aggregated from non-aggregated protein. In experiments involving isolated BAD, the GST tag was removed by treating GSTrap-immobilized BAD-GST with GST-TEV (10:1 ratio, respectively) for 2 h at 4°C. Cleaved protein was removed by washing the column with refolding buffer containing EDTA (0.5 mM). Cleaved BAD was separated from the fusion protein via size exclusion chromatography on a Superdex 200 HR10/30 column at a flow rate of 0.02 mL/min. Protein concentrations were determined from their absorbance at 280 nm using the following extinction coefficients: ε_GCK_ = 32,500 M^-1^cm^-1^, ε_Bcl-xL_ = 41,000 M^-1^cm^-1^, ε_BAD-GST_ = 75,000 M^-1^cm^-1^, ε_BAD_ = 32,000 M^-1^cm^-1^, ε_GST_ = 43,000 M^-1^cm^-1^.

To determine buffer conditions for BAD-GST refolding, we made use of a spin-filter assay developed by Bondos and Bicknell [25]. Purified protein was collected after size-exclusion chromatography and diluted into test buffers containing various stabilizers and detergents, including Triton-X (0.2%), CHAPS (1.25–6.0 mM), arginine (0.1–0.6 M), glycerol (20%) or urea (1.5 M). After overnight incubation at 4°C, diluted protein was filtered across a 100,000 MWCO microconcentrator (Amicon Ultra 0.5, EMD Millipore) at 14,000 x *g*. Concentrate was considered aggregated protein, while flow through was considered non-aggregated protein. Concentrate and flow through were resuspended to equal volumes and analyzed via SDS-PAGE; protein was visualized with SYPRO Ruby stain on a fluorescence imaging scanner. ImageJ software was used to measure ratios of non-aggregated to aggregated protein [26].

### In vitro phosphorylation and phosphomimic variants of BAD

Recombinant BAD-GST was phosphorylated with PKAc after refolding. 1 unit PKAc was added to 10 units BAD-GST and the reaction was dialyzed against BAD refolding buffer (0.1 M Tris, 0.1 M KCl, 0.2 M arginine, 2.5 mM CHAPS, 5 mM DTT, 10% glycerol, pH 8.5) containing ATP (3 mM) and MgCl_2_ (20 mM). Reactions were carried out at 4°C overnight. PKAc and excess ATP were removed from BAD-GST via glutathione-affinity chromatography (GSTrap, GE Healthcare). The extent of phosphorylation was established via mass spectrometry and 2D-IEF-SDS-PAGE using ReadyStrip IPG strips (Bio-Rad) with a pH range of 4–7. pET28*badgst* was subjected to site-directed mutagenesis to remove sites of phosphorylation (Ser-to-Ala) or to incorporate phosphorylation mimics (Ser-to-Asp) using the QuikChange™ Site-directed mutagenesis kit (Stratagene).

### Mass spectrometry

Protein samples were excised from SDS-PAGE gels and exposed to trypsin digestion prior to analysis by mass spectroscopy. MALDI-TOF was performed on a Shimadzu Axima CFP MALDI-TOF in positive ion, reflectron mode. Linear trap quadripole (LTQ) Orbitrap MS was performed on a Thermo LTQ Orbitrap Velos HPLC-nESI-LIT-Orbitrap.

### Size-exclusion chromatography

Size-exclusion chromatography was performed on a HiPrep 16/60 Sephacryl S-200 HR column (GE Healthcare) connected to a Bio-Rad DuoFlow FPLC. The column was calibrated with thyroglobulin (669 kDa), γ-globulin (158 kDa), ovalbumin (43 kDa), myoglobin (17 kDa), and vitamin B12 (1.35 kDa). All proteins were analyzed in BAD buffer (0.1 M Tris, 0.1 M KCl, 0.5 M arginine, 2.5 mM CHAPS, 5 mM TCEP, 10% glycerol, pH 8.5) at a flow rate of 0.02 mL/min at 4°C. Protein samples were concentrated to 800 μL and filtered through 0.2 μm filters before injection onto the size-exclusion column. For complex formation, equimolar mixtures of proteins were added to BAD buffer in a final volume of 800 μL and incubated on ice 30 min prior to injection onto the column.

### SDS-PAGE

Sodium dodecyl sulfate-polyacrylamide gel electrophoresis (SDS-PAGE) analyses were performed using 16.5% denaturing gels prepared according to standard recipes [27]. Precision Plus molecular weight markers (Bio-Rad Laboratories, Inc. Hercules, CA) were used to gauge protein size. Gels were ran at 150 constant volts and stained with colloidal G-250 Coomassie for 30 minutes at room temperature following by destaining overnight in aqueous solutions containing 10% methanol and 10% acetic acid.

### Circular dichroism spectroscopy

Proteins were dialyzed overnight into potassium phosphate buffer (50 mM, pH 7.8) containing KCl (50 mM), glycerol (5% v/v), and TCEP (4 mM). CD spectra were recorded on an AVIV 202 CD spectrometer at 25°C with protein concentrations ranging from 3–15 μM. Spectra were recorded from 200–260 nm in 10 mm pathlength cuvettes using a 1 nm step resolution and 3 s integration time. Three scans were recorded per sample, averaged, and baseline subtracted with appropriate buffer controls. CD signals were converted to molar ellipticity and deconvoluted using CDPro Software.

### Analytical ultracentrifugation

Sedimentation velocity experiments were conducted with an Optima XLI Ultracentrifuge (Beckman Coulter) using a four-hole rotor with three 420 μL samples in standard double-sector Epon centerpieces equipped with sapphire windows. Absorbance and interference data were collected simultaneously. Size-exclusion purified proteins were concentrated to OD_280 nm_ = 0.3–1.0 prior to centrifugation. For complex formation, proteins were mixed in a 1:1 molar ratio in BAD buffer (0.1 M Tris, 0.1 M KCl, 0.5 M arginine, 2.5 mM CHAPS, 5 mM TCEP, 10% glycerol, pH 8.5) and incubated on ice 30 min prior to the start of the experiment. Experiments were carried out at 20°C and 50,000 x *g*. Data were analyzed with Ultrascan III software using a minimum of 70 scans per sample for analysis. Van Holde-Weischet analysis was performed to obtain S_20,W_ values [28].

### Fluorescence polarization assay

A fluorescence polarization assay developed by Zhang, *et al*. was utilized to measure interactions between BAD and its binding partners [29]. Two 21-residue FITC-labeled peptides containing the BH3 domain of BAD were synthesized and purified to >95% by Peptide2.0 (f-BH3 = NLWAAQRYGRELRRMSDK(FITC)FVD; f-pBH3 = NLWAAQRYGRELRRM(p)SDK(FITC) FVD). Polarization was measured at room temperature with an excitation wavelength of 485 nm and an emission wavelength of 530 nm using a SpectraMax M5 microplate reader (Molecular Devices). Experiments to measure binding constants of the fluorescent peptides to Bcl-xL or GCK were performed in black 96-well plates. Each well contained 15 nM peptide, 60 μL BAD buffer (0.1 M Tris, 0.1 M KCl, 0.5 M arginine, 2.5 mM CHAPS, 5 mM TCEP, 10% glycerol, pH 8.5) and variable concentrations of Bcl-xL (0–2.25 μM) or GCK (0–400 μM) in assay buffer (20 mM phosphate buffer, pH 7.4, 1 mM EDTA, 50 mM NaCl, 10 mM DTT) in a final volume of 120 μL. High GCK concentrations were chosen in an effort to detect a signal indicating a binding event. Assays containing GCK also contained 250 mM glucose. Competitive inhibition experiments to measure IC_50_ values of BAD competing with f-BH3 binding to Bcl-xL contained 200 nM f-BH3, 50 nM Bcl-xL, and 60 μL BAD (0–50 μM) in a final volume of 120 μL. Plates were incubated at room temperature in the dark for 15 min prior to polarization measurements.

### Isothermal titration calorimetry

Isothermal titration calorimetry was performed at 25°C using a MicroCal VP-ITC (GE Healthcare) instrument. Bcl-xL (100 μM) in BAD buffer (0.1 M Tris, 0.1 M KCl, 0.5 M arginine, 2.5 mM CHAPS, 5 mM TCEP, 10% glycerol, pH 8.5) was titrated into the calorimeter cell containing size-exclusion chromatography purified BAD-GST (10–18 μM) in 4 μL injections. A 21-residue phosphorylated peptide containing the BH3 domain of BAD was synthesized and purified to >95% by Peptide2.0 (pBH3 = NLWAAQRYGRELRRM(p)SDKFVD). The peptide (300 μM) in potassium phosphate buffer (25 mM) containing KCl (25 mM), TCEP (4 mM) and DMSO (5%) with and without glucose (200 mM) was titrated into the cell containing GCK (10 μM) previously dialyzed against the same buffer. GCK (100 μM) in BAD buffer was titrated into the cell containing S118D BAD-GST (10–15 μM) in 13 μL injections. Data were analyzed using Origin software.

### Chemical cross-linking of BAD and glucokinase

Protein samples were cross-linked with dimethyl pimelimidate (DMP). Aqueous stocks of cross-linkers were added to protein samples in a 50-fold molar excess and incubated at room temperature 1 h. Mixtures contained 20 μM protein (GCK or BAD) or 40 μM protein (1:1 mixture of glucokinase and BAD) in BAD buffer (0.1 M Tris, 0.1 M KCl, 0.5 M arginine, 2.5 mM CHAPS, 5 mM TCEP, 10% glycerol, pH 8.5). Reactions were immediately separated on SDS-PAGE and visualized with Coomassie blue staining.

### Kinetic characterization of glucokinase

GCK activity was assayed using a glucose 6-phosphate dehydrogenase (G6PDH)-linked assay at 25°C. 50 nM GCK was assayed in buffer containing HEPES (100 mM, pH 7.4), KCl (50 mM), NADP^+^ (0.5 mM), DTT (10 mM), MgCl_2_ (7 mM), G6PDH (1.5 U) and glucose (0–100 mM) in a total volume of 94 μL. Addition of ATP (6 mM) initiated the reaction. Production of NADPH was measured at 340 nm. To determine the effect of BAD on GCK activity, 5 μL of BAD or buffer control was added to the reaction in a final volume of 100 μL. *K*_0.5_ values were determined by fitting to the Hill equation. The effect of BAD on GCK activity is reported as the percent rate increase at individual glucose concentrations, calculated as the average of a minimum of four rates of BAD-containing assays divided by the average of rates of BAD buffer-containing assays. The results represent the average of at least three independent preparations of pure BAD.

Steady state kinetics were also performed as described by Szlyk, *et al*.[13]. Briefly, G6PDH-linked assays were performed in clear 96-well plates at 37°C with a SpectraMax M5 microplate reader (Molecular Devices) recording absorbance at 340 nm every 30 s. Reaction mixtures contained GCK (7.5 nM), HEPES (100 mM, pH 7.4), KCl (150 mM), MgCl_2_(6 mM), DTT (1 mM), NAD (1 mM), BSA (0.05%), G6PDH (2.5 U), glucose (0.5–100 mM), and ATP (5 mM) in the presence or absence of BAD-GST S118D variant (5 μM) or phosphorylated BAD BH3 peptide (5 μM) in a final volume of 100 μL.

## Results

### Production and purification of recombinant human BAD

To facilitate biochemical characterization of the BAD:GCK complex, we sought a method to produce functional, recombinant human BAD. Several groups have used microbial hosts to produce BAD, however, functional characterization of the resulting protein was limited. Hinds *et al*. report that recombinant BAD produced in *Escherichia coli* is highly susceptible to proteolysis [30]. These investigators also found that recombinant BAD eluted from size-exclusion columns with a larger than expected apparent molecular mass. Consistent with these observations, we found that production of recombinant BAD under native conditions leads to aggregation of the protein. We also observed extensive C-terminal proteolysis of recombinant BAD, as revealed by MALDI-TOF analysis. Addition of protease inhibitors failed to alleviate degradation. To combat these issues, we analyzed the primary sequence of BAD using the PONDR algorithm and found that nearly 80% of residues in BAD are predicted to be unstructured [31–33]. Based on these results, we pursued a purification procedure that utilizes denaturing conditions, a strategy that has proven successful for other disordered proteins [34,35].

**Fig 1:**
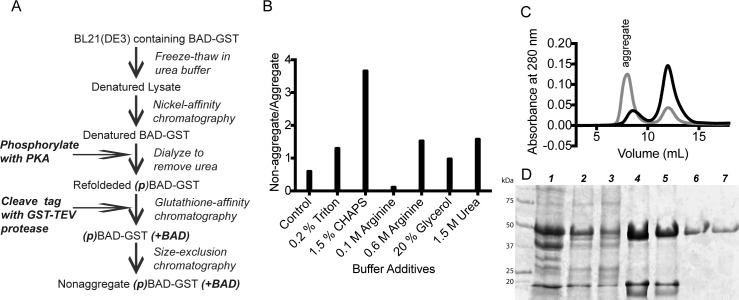
Development of a protocol for production and purification of recombinant BAD-GST. (A) Overview of the optimized protocol for BAD-GST production. (B) Impact of refolding buffer additives upon BAD-GST aggregation, as evaluated by the spin-filter binding assay developed by Bondos [25]. (C) Size-exclusion chromatogram of HisTrap purified BAD-GST in the absence (gray) and presence (black) of arginine (0.5 M) and CHAPS (2.5 mM). (D) Coomassie blue stained SDS-PAGE of BAD-GST throughout the purification protocol. *Lane 1*: Insoluble fraction following cell lysis; *Lane 2*: Soluble fraction; *Lane 3*: Ni-NTA affinity column flow through; *Lane 4*: Ni-NTA affinity column eluate; *Lane 5*: glutathione-affinity column flow through; *Lane 6*: glutathione-affinity column eluate; *Lane 7*: Size-exclusion purified BAD-GST.

Following extensive testing of experimental expression and purification methods, we identified an optimal protocol for producing recombinant BAD as a C-terminal GST fusion polypeptide in *E*. *coli* using a T7-based expression system (Fig 1). Maximal expression of BAD-GST is observed in cells grown at 37°C and induced with low IPTG concentrations during the pre-exponential growth phase. Following a brief induction period, harvested cells are lysed in buffer containing 6 M urea. Unfolded BAD-GST is purified via immobilized metal affinity chromatography and refolded via sequential dialysis to remove denaturant. We found that the presence of two additives, CHAPS (2.5 mM) and arginine (0.5 M), was essential to prevent aggregation of recombinant BAD (Fig 1B and 1C). Subsequent glutathione affinity chromatography affords correctly folded protein, and a final size-exclusion chromatographic step is performed to isolate non-aggregated, functional BAD. This protocol routinely yields 0.5 mg of BAD per liter of cell culture, with a purity >95% based on SDS-PAGE analysis (Fig 1).

### Structural characterization of recombinant BAD

**Fig 2:**
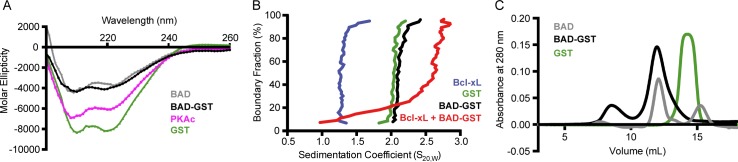
Characterization of recombinant BAD-GST. (A) CD spectra of recombinant BAD-GST (black), BAD following cleavage and removal of the GST tag (gray), BAD-GST following *in vitro* phosphorylation with PKAc (purple) and GST (green). (B) Analytical ultracentrifugation sedimentation velocity analysis of BAD-GST (black), GST (green), Bcl-xL (blue) and a 1:1 molar mixture of BAD-GST with Bcl-xL (red). (C) Size-exclusion chromatogram of BAD-GST (black), BAD following cleavage and removal of the GST tag (gray), and GST (green).

The PONDR algorithm predicts that BAD lacks extensive secondary structure [31–33]. To test this prediction, we used far UV circular dichroism spectroscopy to investigate the structure of the BAD-GST fusion polypeptide. Recombinant BAD-GST contains 37% β-sheet and 10% α-helical character. Unexpectedly, only 30% of the structure exists as random coil (Fig 2A). By comparison, GST is 24% β-sheet, 25% α-helical and 30% random coil. To eliminate the possibility that GST influences the secondary structure of BAD, we performed CD spectroscopy following proteolytic cleavage and removal of the GST tag. The CD spectra of BAD and BAD-GST are nearly identical, suggesting that GST does not significantly alter the secondary structure of BAD. To compare these results with another Bcl-2 family member, we determined the CD spectrum of recombinant human Bcl-xL. As expected, Bcl-xL has a high degree of helical content (43%) and a small amount of β-sheet (14%) (data not shown). The overall secondary structural content of BAD and Bcl-xL determined in these studies are similar, with roughly 30% of each protein adopting a random coil conformation.

To investigate the oligomerization state of recombinant BAD-GST, we conducted analytical ultracentrifugation (AUC) sedimentation velocity experiments (Fig 2B). AUC is a robust method of determining protein molecular weight, and it can estimate the compactness of a biomolecule from the frictional ratio value (f/f_0_). Compact spheres posses f/f_0_ of unity, and larger values indicate more elongated or asymmetric conformations. The predicted molecular weight of the BAD-GST monomer is 47 kDa, and the molecular weight of BAD-GST determined from sedimentation velocity experiments is 98 kDa. This observation is consistent with the established ability of GST to form homodimers. AUC sedimentation velocity experiments on isolated GST reveals a molecular weight of 57 kDa, confirming that GST dimerizes under our experimental conditions. BAD-GST displays an f/f_0_ value of 3.9, indicating that the protein exists as a rod-shaped dimer. By comparison, the f/f_0_ value of GST is 2.4, as expected for a globular dimer. The results of our AUC analyses confirm that BAD-GST is not aggregated, and the data are consistent with the relative migration rates of BAD-GST and GST observed during preparative-scale size-exclusion chromatography (Fig 2C).

### Phosphorylation of recombinant BAD

The physiological activity of human BAD, including its ability to interact with known binding partners, is regulated by phosphorylation of three serine residues located at positions 75, 99, 118 [36]. We found that incubation of BAD-GST with PKA is sufficient to achieve phosphorylation at all three sites, as verified by LTQ Orbitrap mass spectrometry. Isoelectric focusing revealed four distinct populations of phosphorylated BAD-GST with pI values ranging from 4–7. We also created serine-to-aspartate variants at positions 75, 99, and 188 to act as functional phosphomimics of modified BAD-GST. We observed that phosphorylated BAD-GST and individual phosphomimic variants resulted in higher yields of monomeric protein compared to non-phosphorylated BAD-GST. To explore the impact of phosphorylation on the structural characteristics of recombinant BAD-GST, we utilized circular dichroism spectroscopy and analytical ultracentrifugation. CD spectroscopy indicates that phosphorylated BAD-GST displays a higher degree of α-helical content compared to unmodified BAD-GST (Fig 2A), and AUC reveals that phosphorylation decreases the f/f_0_ from 3.9 to 3.1. These data suggest that phosphorylation of BAD results in a more structured protein.

## Functional characterization of recombinant BAD

**Fig 3:**
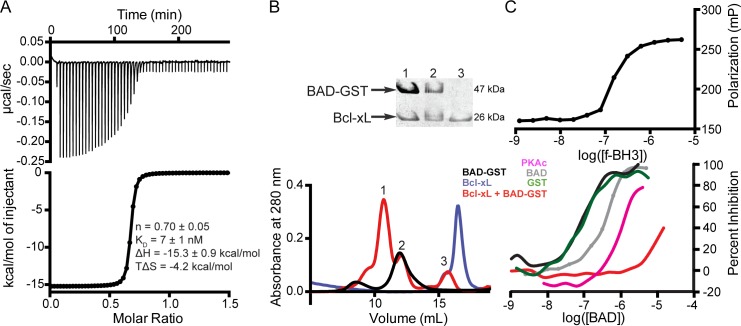
Functional assays of recombinant BAD-GST. (A) Representative isotherm for the interaction of BAD-GST and Bcl-xL. The thermodynamic parameters represent the average of two independent experiments. (B) Size exclusion chromatogram of BAD-GST (black), Bcl-xL (blue) and a 1:1 mixture of BAD-GST and Bcl-xL (red). Coomassie blue stained SDS-PAGE analysis of individual fractions (above), confirms formation of a higher molecular weight complex between BAD-GST and Bcl-xL. (C) Fluorescence polarization assays demonstrate that f-BH3 binding to Bcl-xL (top) is disrupted by recombinant BAD-GST (black) or BAD following cleavage and removal of the GST tag (gray), with IC_50_ values of 130 nM and 440 nM, respectively. Phosphorylation of BAD-GST by PKA (pink) abrogrates the protein’s ability to compete with f-BH3. Phosphomimetics demonstrate that phosphorylation at S118 (red), but not S75 and S99 (green), is responsible for impaired binding to Bcl-xL.

The functionality of recombinant BAD-GST was assessed by testing its ability to interact with a known binding partner, Bcl-xL. Soluble, recombinant Bcl-xL was produced via a previously described protocol [23], and the interaction between Bcl-xL and BAD-GST was monitored using isothermal titration calorimetry. Our data indicate that the interaction between BAD-GST and Bcl-xL is characterized by a high affinity (*K*_d_ = 7 nM) and a favorable change in enthalpy (Fig 3A). Size exclusion chromatographic analysis of a 1:1 mixture of purified Bcl-xL and BAD-GST reveals three peaks corresponding to monomeric Bcl-xL, the BAD-GST dimer, and the BAD-GST:Bcl-xL complex (Fig 3B). Analytical ultracentrifugation sedimentation velocity analysis performed on this mixture produced sedimentation coefficients corresponding to monomeric Bcl-xL (1.3), the BAD-GST dimer (2.1) and the BAD-GST:Bcl-xL complex (2.6) (Fig 2B). The BAD-GST:Bcl-xL complex has an experimentally determined molecular weight of 150 kDa, which equals the BAD-GST dimer bound to two molecules of Bcl-xL. Thus, both BAD subunits of the dimer are available to interact with Bcl-xL.

To investigate the impact of BAD-GST modifications on its association with Bcl-xL, we utilized an established binding assay in which a fluorescently labeled BH3 peptide (f-BH3) experiences an increase in polarization upon binding to Bcl-xL [29]. We determined a *K*_d_ value of 180 nM describing the association of f-BH3 to Bcl-xL, consistent with past reports (Fig 3C, top)[37,38]. BAD-GST competes with f-BH3 binding to Bcl-xL, with an IC_50_ value of 130 nM (Fig 3C). Removal of the GST tag increases the IC_50_ value of BAD binding to Bcl-xL to 440 nM (Fig 3C). We attribute this increase in IC_50_ value to the reduced stability and increased aggregation propensity of isolated BAD. GST alone at concentrations up to 10 μM does not compete with f-BH3 binding to Bcl-xL. Phosphorylation of BAD-GST greatly reduces the protein’s ability to compete with f-BH3, resulting in a lower limit of 1.6 μM for the IC_50_ value. To determine which phosphorylation site(s) impact binding, we produced aspartate variants to mimic the phosphoserines. The S75D-S99D-S118D triple variant of BAD-GST exhibits an IC_50_ value greater than 19 μM. The S75D-S99D double variant displays an IC_50_ value of 87 nM, and the S118D variant exhibits an IC_50_ value exceeding 15 μM, confirming the importance of residue S118 for the BAD:Bcl-xL interaction. Together, our size-exclusion chromatographic, analytical ultracentrifugation, isothermal titration calorimetry, and fluorescence polarization data provide compelling evidence that recombinant BAD-GST is functional.

### Interaction of recombinant BAD and BAD BH3 peptides with human GCK

**Fig 4:**
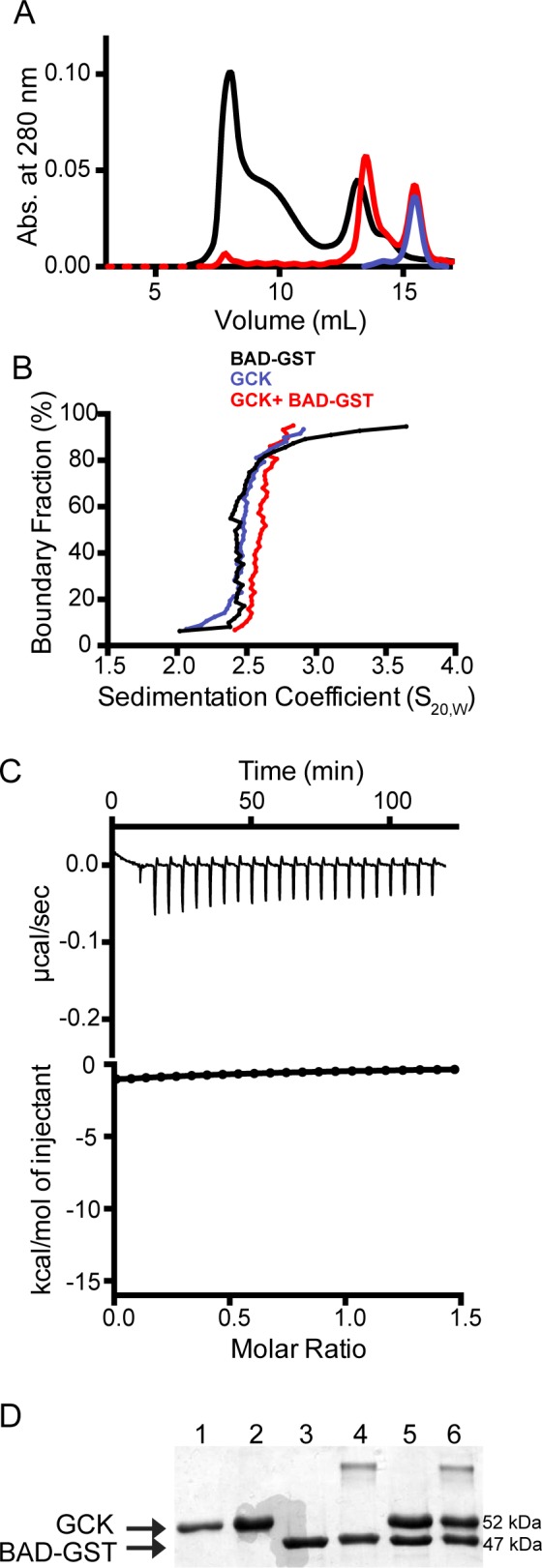
Characterization of the interaction between GCK and BAD-GST. (A) Size-exclusion chromatogram of an equimolar mixture of BAD-GST and GCK (red) overlaid with BAD-GST (black) and GCK (blue) provides no evidence of complex formation. (B) Analytical ultracentrifugation sedimentation analysis of an equimolar mixture of BAD-GST and GCK (red) overlaid with BAD-GST (black) and GCK (blue) does not reveal a higher molecular weight component indicative of complex formation. (C) Isotherm for the addition of recombinant S118D BAD-GST to GCK. (D) Coomassie blue stained SDS-PAGE analysis of mixtures of BAD-GST and GCK following cross-linking with dimethyl pimelimidate (DMP). Lane 1: GCK; Lane 2: GCK + DMP; Lane 3: phosphorylated BAD-GST; Lane 4: phosphorylated BAD-GST + DMP; Lane 5: equimolar mixture of GCK and phosphorylated BAD-GST; Lane 6: equimolar mixture of GCK and phosphorylated BAD-GST + DMP. The higher molecular weight bands in lanes 4 and 6 result from GST dimerization.

A variety of biochemical methods were employed to characterize the interaction of BAD-GST with human pancreatic GCK. Size-exclusion chromatographic analyses of mixtures of BAD-GST and GCK across a range of protein concentrations reveal two peaks corresponding to dimeric BAD-GST and monomeric GCK (Fig 4A). No evidence of a higher molecular weight complex is observed. Analytical ultracentifugation sedimentation velocity experiments conducted on equimolar mixtures of phosphorylated BAD-GST and GCK show no evidence of a higher molecular weight complex (Fig 4B). Isothermal titration calorimetry revealed no detectable binding event when GCK was added to a solution of the S118D BAD-GST variant, a protein expected to show the highest affinity for interaction with GCK (Fig 4C) [8,13]. These data contrast with the results of size-exclusion chromatographic, AUC, and ITC analyses of BAD-GST and Bcl-xL mixtures, which provide clear evidence of complex formation. Chemical cross-linking also does not support the formation of a BAD-GST:GCK complex (Fig 4D). Notably, GST and BAD-GST homodimers are visualized following cross-linker treatment, providing a positive control for this experimental method. Addition of GCK does not interfere with the formation of cross-linked BAD-GST dimers.

**Fig 5:**
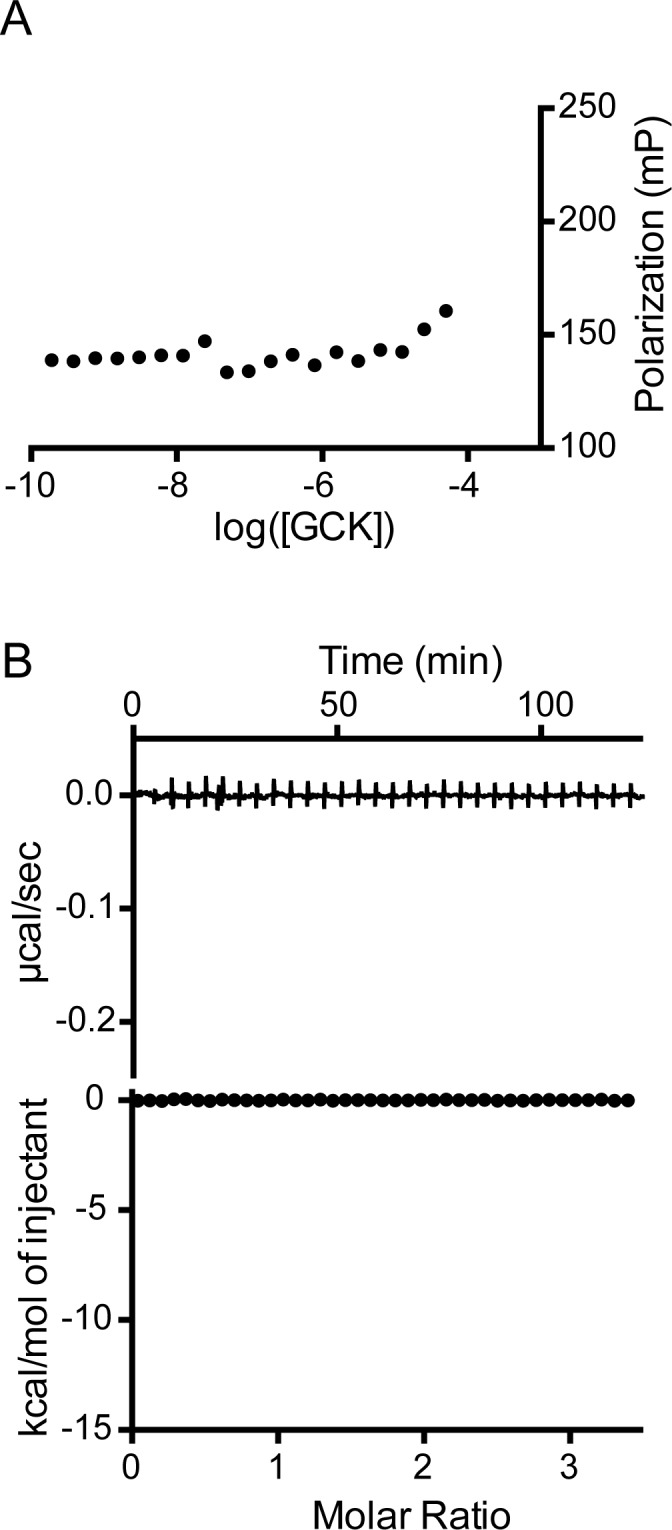
Characterization of the interaction between GCK and peptide analogs of the BAD BH3 domain. (A) GCK at concentrations up to 10 μM does not increase the fluorescence polarization of f-BH3; (B) Isotherms for the addition of phosphorylated BH3 peptide to GCK do not indicate an interaction.

Stapled peptide analogs modeled after the BAD BH3 domain reportedly interact with GCK directly [8,13]. Based on this observation, we investigated the ability of unmodified and phosphorylated BAD BH3 peptides to associate with GCK via two different methods. We incubated GCK with the fluorescent BH3 peptide (f-BH3) previously employed to quantify the association of BAD-GST with Bcl-xL. No change in fluorescence polarization of the f-BH3 peptide is observed across a range of GCK concentrations (10^−9^ to 10^−5^ M), both in the presence and absence of 0.2 M glucose (Fig 5A). At very high GCK concentrations, a small, non-saturable increase in polarization is observed. We attribute this signal to crowding, since a similar increase in polarization is observed when high concentrations of BAD-GST are added to the peptide. Phosphorylation of f-BH3 at the position corresponding to Ser-118, as well as addition of trifluoroethanol (20–40%), which induces peptide secondary structure, fails to promote binding to GCK (S1 Fig). We also performed isothermal titration calorimetric binding assays in which a non-fluorescent BH3 peptide bearing a phosphorylated serine at position 118 was titrated into purified GCK (10 μM). No detectable heat change is observed upon addition of the phosphorylated peptide at concentrations up to 50 μM, both in the presence and absence of 0.2 M glucose (Fig 5B).

### Impact of recombinant BAD and BAD BH3 peptides on GCK activity

**Fig 6:**
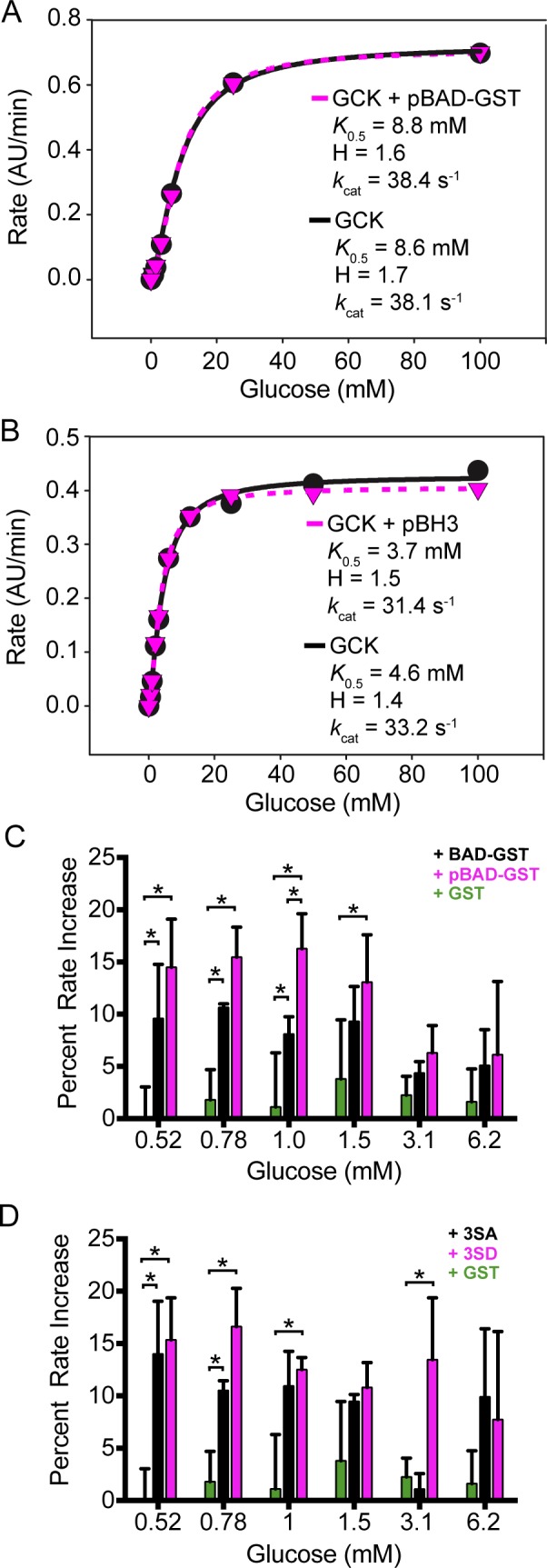
Impact of recombinant BAD and BAD BH3 peptides on GCK activity. GCK kinetic constants are unaffected by (A) phosphorylated BAD-GST or (B) a phosphorylated BH3 analog. (C) GCK activity is modestly stimulated by BAD-GST (black) or phosphorylated BAD-GST (purple) compared with GST (green) at glucose concentrations below the *K*_0.5_ value. (D) GCK activity is stimulated equally well by the 3SA (black) and 3SD (purple) variants of BAD-GST compared with GST (green). * indicates p < 0.05.

Stapled peptide analogs modeled after the BAD BH3 domain reportedly increase the *V*_max_ value of GCK by 40% [13]. We investigated the ability of a phosphorylated BAD BH3 peptide to alter the kinetic constants of GCK using an established spectrophotometric assay routinely employed in our lab [18,39–41]. Addition of peptide at concentrations up to 5 μM does not impact the *k*_cat_, *K*_0.5_ or Hill coefficient of human GCK (Fig 6A). To account for differences in experimental conditions between these assays and past studies, we also performed microplate-based assays using a protocol identical to that employed by Szlyk *et al*. [13]. We observed no statistically significant difference in the GCK *V*_max_ value using either assay (Fig 6A and S2 Fig).

To explore the extent to which full-length BAD-GST impacts the kinetic characteristics of human GCK, we determined the steady-state kinetic profile of GCK in the presence and absence of BAD-GST at variable glucose concentrations. The presence of BAD-GST (8 μM) does not alter the *k*_cat_, *K*_0.5_ or Hill coefficient of GCK (Fig 6B). These experiments reveal modest increases in GCK activity at glucose concentrations below the *K*_0.5_ value. A systematic investigation demonstrates that BAD-GST stimulates GCK activity to a small, but statistically significant extent at glucose concentrations ranging from 0.5–1.0 mM (Fig 6C). The largest increase in activity is observed at 0.8 mM glucose, where addition of BAD-GST (≥ 2 μM) produces a 10% increase in rate. Similar effects are observed when the GST tag is removed from BAD; GCK activity increases 7–12% at glucose concentrations of 0.5, 0.78 and 1.0 mM (data not shown).

To determine if phosphorylation of BAD increases its ability to stimulate GCK activity, as previously reported, BAD-GST was phosphorylated *in vitro* with recombinant PKA. Phosphorylated BAD-GST increases GCK activity 5% over unmodified BAD-GST at 1.0 mM glucose (Fig 6C). At all other glucose concentrations there is no statistical difference in the effect of phosphorylated versus unmodified BAD-GST on GCK activity. After cleavage of the GST tag, phosphorylated BAD increases GCK activity to a similar extent as unmodified BAD. We utilized the S75D-S99D-S118D triple variant of BAD-GST (3SD) to mimic a homogenous fully phosphorylated protein. As a negative control, we also utilized a variant in which alanine was substituted at the three target serine residues (3SA). Both the 3SD and 3SA BAD-GST variants increase GCK activity to the same relative extent as wild-type BAD-GST (Fig 6D), but only at glucose concentrations below the *K*_0.5_ value. Notably, GST alone has no mpact on enzyme activity under all conditions tested.

## Discussion

To date, most investigations of BAD function have focused on the BH3 domain. This region governs BAD’s ability to interact with a variety of binding partners. *In vitro* functional studies of the protein commonly employ synthetic peptides that encompass only 15 to 25 residues within BAD’s canonical BH3 domain [8,13,29,38]. The limited scope of these investigations stems, in part, from an inability to produce full-length human BAD in the laboratory. Here, we present a method for the production and purification of functional human BAD in *Escherichia coli*. Importantly, our experimental procedure provides sufficient quantities of full-length BAD for a variety of downstream biochemical and biophysical investigations, many of which have been intractable until now. The development of a method for recombinant BAD production is expected to facilitate a variety of new investigations into the structure and function of this important human protein.

Characterization of recombinant BAD reveals a folded, non-aggregated protein that recapitulates the known functional properties of this protein, including its ability to interact with human Bcl-xL in a phosphorylation dependent manner. Isothermal titration calorimetry demonstrates that the interaction between Bcl-xL and full-length BAD is enthalpically driven, a finding that is consistent with the extensive interaction surface observed in crystal structures of Bcl-xL bound to BH3 peptides [23,38,42]. The dissociation constant of the full-length BAD:Bcl-xL complex determined by our ITC experiments is nearly identical to previously reported values for the interaction of Bcl-xL with BAD BH3 peptide analogs [29,37]. This observation indicates that regions outside of the BH3 domain do not contribute significantly to the ability of BAD to interact with Bcl-xL. Phosphorylation of recombinant BAD abrogates binding to Bcl-xL, and functional characterization of various recombinant phosphomimic variants confirm that modification at Ser118 within the BH3 domain is solely responsible for decreased affinity toward Bcl-xL. These functional characteristics are consistent with literature precedent and indicate that recombinant BAD is equivalent to the native protein in terms of its ability to interact with a known binding partner [10,38].

Phosphorylation at Ser118 dictates BAD’s ability to impact glucose metabolism in mouse islets and this modification appears to play a central role in governing affinity for GCK [8,43]. Despite this fact, we detected no interaction between GCK and recombinant, full-length BAD following *in vitro* phosphorylation by PKA. We also detected no interaction between GCK and a phosphomimic version of full-length BAD or a phosphorylated BH3 peptide. ITC and fluorescence polarization assays confirmed that phosphorylated recombinant BAD, the S118D variant and a phosphorylated BH3 peptide were each significantly impaired in their ability to interact with Bcl-xL. Thus, the lack of a phosphorylation-dependent interaction of BAD with GCK cannot be explained by insufficient phosphorylation within the BH3 domain.

We turned to enzymatic assays to investigate the functional impact of BAD upon GCK activity. We reasoned that enzymatic assays might provide sufficient sensitivity to observe a functionally significant protein-protein interaction in light of observations that phosphorylated analogs of BAD’s BH3 domain increase the *V*_max_ value of GCK by up to 44% [13]. This level of activation should be readily detectable using the GCK activity assay commonly employed in our lab. In our hands, however, neither native nor phosphorylated versions of full-length recombinant BAD produced measurable increases in the *V*_max_ value of GCK. We observed small increases in GCK activity at glucose concentrations below the *K*_0.5_ value, which reached a maximum value of 15% when GCK was assayed in the presence of recombinant phosphorylated BAD (> 2 μM). A comparable degree of activation was observed at 0.78 mM when GCK was assayed in the presence of the S75D-S99D-S118D triple variant (> 2 μM). The physiological relevance of BAD-mediated GCK activation is questionable given the magnitude of the stimulatory effect observed in these studies and the high concentration of BAD required for their observation.

Perhaps the most perplexing result from this study is the lack of an increase in the GCK *V*_max_ value in the presence of a phosphorylated BH3 peptide. This peptide is the same length and contains the same amino acid sequence as the stapled peptide analog previously used to characterize the GCK-BAD interaction [8,13]. The only difference between the two peptides is the presence of a hydrocarbon bridge and a benzophenone moiety in the original stapled peptide. The benzophenone moiety was incorporated to facilitate crosslinking between the peptide and binding partners, and the hydrocarbon stable was installed to stabilize α-helical structure. In principle, the increased secondary structural content provided by the staple could favorably contribute to the interaction with GCK; however, the apparent binding affinity of the stapled peptide for Bcl-xL differs by only 5-fold from the binding affinity determined for our unmodified BH3 peptide binding to Bcl-xL. Prior spectroscopic studies indicate that the hydrocarbon staple increases net α-helical content by ~ 2-fold [8]. Thus, the impact on binding affinity from the increased helical content of the peptide as a result of the staple is expected to be 10-fold or less. We observed no change in GCK activity at peptides concentrations up to 50 μM. In contrast, Szylk *et al*. reported that stimulatory attributes of stapled BH3 peptides reached saturation near 5 μM [13]. Moreover, the addition of trifluoroethanol, which increases the helicity of peptides, had no effect upon the ability of peptides to alter GCK activity. Together, these results cast uncertainty upon the specificity and mechanism of action of stapled peptide analogs that reportedly stimulate GCK and promote insulin release in BAD-deficient islets [8]. It seems possible that the activating properties of these analogs could result from the presence of non-natural chemical moieties within the peptide, rather than the constituent amino acids themselves.

The first indication of a putative interaction between full-length BAD and GCK came from the observation that both proteins co-localize with one another in a macromolecular complex purified from mouse hepatocyte mitochondrial extracts [7]. Support of a BAD:GCK interaction was provided by the observation that BAD co-immunoprecipitates with GCK following *in vitro* transcription-translation of both proteins in rabbit reticulocyte lysates [8]. In contrast to these experiments, our study utilizes active, highly purified homogeneous preparations of both proteins. The biochemical and biophysical experiments described herein provide a direct test for interaction and our data provide evidence that GCK and BAD do not associate with one another to an appreciable extent *in vitro*. The lack of a detectable direct interaction between full-length BAD and human GCK suggests that association of these two proteins within cells requires the presence of an additional factor or factors. Likely candidates for such a factor include the other components of the mitochondrial complex such as PKA, PP1 or WAVE-1; however, these proteins are not expected to be present in high concentrations in rabbit reticulocytes. Thus, they are unlikely to explain the observation of a detectable BAD:GCK interaction following *in vitro* transcription-translation.

In conclusion, we have developed a method for production of recombinant human BAD that should enable a variety of experimental investigations of this protein. We have demonstrated that highly purified preparations of recombinant BAD are incapable of interacting with GCK using a variety of *in vitro* methods. We also demonstrate that GCK activity is largely unaffected by the presence of both full-length BAD and peptide analogs of the protein’s BH3 domain. Our data support the postulate that one or more additional factors are required to promote the interaction of GCK and BAD. The identity of these putative factors remains an important unanswered question, which warrants further investigation.

## Supporting information

S1 Fig Alpha-helix inducing agent does not affect f-pBH3 binding to GCK. Polarization of 34 μM f-pBH3 by increasing concentrations of GCK was assayed in the presence of 40% triflouroethanol.
(EPS)

S2 Fig Buffer conditions used by Szlyk *et al*. did not increase BAD’s effects on GCK kinetics. GCK (7.5 nM) was assayed in the presence of A. S118D BADGST (5 μM) or B. pBH3 (5 μM) in a 96 well plate using a microplate reader. Buffer conditions were as follows: Ref [13]: 100 mM Hepes, pH 7.4; 150 mM KCl; 1 mM NAD+; 0.05% BSA; 6 mM MgCl_2_; 1 mM DTT; 2.5 U G6PDH; 5 mM ATP. Optimal GCK conditions: 100 mM Hepes, pH 7.4; 150 mM KCl; 1 mM NADP+; 6 mM MgCl_2_; 10 mM DTT; 1.5 U G6PDH; 5 mM ATP.
(EPS)
